# Sulfaquinoxaline Oxidation and Toxicity Reduction by Photo-Fenton Process

**DOI:** 10.3390/ijerph18031005

**Published:** 2021-01-23

**Authors:** Vanessa Ribeiro Urbano, Milena Guedes Maniero, José Roberto Guimarães, Luis J. del Valle, Montserrat Pérez-Moya

**Affiliations:** 1 Chemical Engineering Department, Universitat Politècnica de Catalunya, Escola d’Enginyeria de Barcelona Est (EEBE), Av. Eduard Maristany, 16, 08019 Barcelona, Spain; nessaru@gmail.com (V.R.U.); luis.javier.del.valle@upc.edu (L.J.d.V.); 2 School of Civil Engineering, Architecture and Urban Design, FEC, University of Campinas, Unicamp, P.O. Box 6143, Campinas 13083-889, Brazil; milenaguedesmaniero@gmail.com; 3 Barcelona Research Center for Multiscale Science and Engineering, Universitat Politècnica de Catalunya, Escola d’Enginyeria de Barcelona Est (EEBE), 08019 Barcelona, Spain

**Keywords:** advanced oxidation process, photo-Fenton, emerging contaminants, sulfaquinoxaline, experimental design, oxidations, mineralization, toxicity

## Abstract

Sulfaquinoxaline (SQX) has been detected in environmental water samples, where its side effects are still unknown. To the best of our knowledge, its oxidation by Fenton and photo-Fenton processes has not been previously reported. In this study, SQX oxidation, mineralization, and toxicity (*Escherichia coli* and *Staphylococcus aureus* bacteria) were evaluated at two different setups: laboratory bench (2 L) and pilot plant (15 L). The experimental design was used to assess the influence of the presence or absence of radiation source, as well as different H_2_O_2_ concentrations (94.1 to 261.9 mg L^−1^). The experimental conditions of both setups were: SQX = 25 mg L^−1^, Fe(II) = 10 mg L^−1^, pH 2.8 ± 0.1. Fenton and photo-Fenton were suitable for SQX oxidation and experiments resulted in higher SQX mineralization than reported in the literature. For both setups, the best process was the photo-Fenton (178.0 mg L^−1^ H_2_O_2_), for which over 90% of SQX was removed, over 50% mineralization, and bacterial growth inhibition less than 13%. In both set-ups, the presence or absence of radiation was equally important for sulfaquinoxaline oxidation; however, the degradation rates at the pilot plant were between two to four times higher than the obtained at the laboratory bench.

## 1. Introduction

Pharmaceutical products present different behaviors in the environment, with rates of persistence, dispersion, and accumulation depending on each compound specifically. However, even at trace concentrations, these compounds can significantly affect the biosphere, due to their high biological activity [1], with impacts on non-target species.

The release of pharmaceuticals (parent compounds and/or metabolites) through excretion is one of the main routes of these compounds to the environment [2]. These substances are commonly detected in groundwater [3], surface water [4], and mineral water [5], mainly because wastewater treatment plants are not capable of degrading them [6,7].

Sulfaquinoxaline (SQX), a sulfonamide class compound, is one of the oldest synthetic antimicrobials still used, mainly in veterinary medicine, due to its low cost and broad-spectrum activity against Gram-negative and Gram-positive bacteria and protozoa, in order to prevent coccidiosis and bacterial infections [8,9]. It is also used in rodenticides because it acts as an anticoagulant, due to the inhibition of K vitamin synthesis [10]. This compound shows low adsorption on soil (log K_ow_ = 1.7) and as a result, it has been detected in environmental waters at concentrations ranging from 29.5 to 40.8 ng L^−1^ [4]. As the effects of this contaminant in the environment are not completely known, sulfaquinoxaline is considered a contaminant of emerging concern.

Advanced oxidation processes (AOPs) are a technology able to remove contaminants of emerging concern from water by reactions involving the hydroxyl radicals (HO^•^), which have a high reduction potential (2.8 V). The current applications of AOPs, such as Fenton, ozonation, photocatalytic oxidation, electrochemical oxidation and so on, for the degradation of antibiotics in water and wastewater have been analyzed and summarized in several recent publications [11,12]. The knowledge around degradation of antibiotics have made some progress, however future research is required and still many aspects are under discussion.

AOPs can be used in preliminary or complementary treatments associated with conventional treatment systems. It is important to consider that the degradation products formed after the HO^•^ reaction can present similar or even higher biological activity than the parent compound [13]. For this reason, toxicity assays are essential to evaluate the biological activity of the compound studied as well as the degradation products formed during the process.

The Fenton technique is an advanced oxidation process that generates HO^•^ from hydrogen peroxide decomposition catalyzed by the presence of ferrous ions in solution (Equation (1)). The Fenton reaction is fast, but the process becomes slower when the Fe(II) is oxidized to Fe(III), resulting in a slower production of HO^•^ (Equation (2)).

Faster regeneration of Fe(II) from Fe(III) (Equation (3)) occurs in the photo-Fenton process, increasing HO^•^ formation and a higher degradation rate, compared to the Fenton process. In addition, competitive reactions can occur when the Fenton reagents are present in large excess, scavenging the oxidant species (Equations (4)–(6)) [14,15,16,17].

(1) $$Fe\left(II\right)+H_{2}O_{2}\to Fe\left(III\right)+HO^{•}+OH^{-}\;k\;=\;70\;{\mathrm{mol}}^{-1}\;{L\;s}^{-1}$$

(2) $$Fe\left(III\right)+H_{2}O_{2}\to Fe\left(II\right)+HO_{2}^{•}+H^{+}\;k\;=\;0.001\;–\;0.01\;{\mathrm{mol}}^{-1}\;{L\;s}^{-1}$$

(3) $$FeOH^{2+}+hv\to Fe\left(II\right)+HO^{•}$$

(4) $$HO^{•}+Fe\left(II\right)\to Fe\left(III\right)+OH^{-}$$

(5) $$HO^{•}+H_{2}O_{2}\to HO_{2\;}^{•}+H_{2}O$$

(6) $$HO^{•}+HO^{•}\to H_{2}O_{2}$$

Fenton and photo-Fenton processes have been shown to be suitable technologies for the oxidation of contaminants of emerging concern, including quinolones [18,19], metronidazole [20], other antibiotics, and anti-inflammatories [21].

Sulfaquinoxaline oxidation was previously reported by Liao et al. [22], who used the UV/H_2_O_2_ process; by Qiu et al. [23], who studied the oxidation of sulfonamides during the aerobic composting of animal manures; by Ghanem et al. [24], who studied hydrolysis, photolytic, and thermal degradation processes; and by Urbano et al. [25,26], who studied sulfaquinoxaline oxidation by photoperoxidation and ozonation processes. However, to the best of the authors’ knowledge, there have been no previous reports of sulfaquinoxaline oxidation by Fenton and photo-Fenton processes, with the determination of the residual toxicity, although these processes seem to be efficient in removing other sulfonamides from water matrices [27,28,29].

The objective of this study was to evaluate the efficiency of Fenton and photo-Fenton processes for sulfaquinoxaline oxidation and mineralization using two different setups: laboratory bench (2 L) and pilot plant (15 L) and to monitor the residual toxicity of the solutions submitted to these oxidation processes using the *Escherichia coli* and *Staphylococcus aureus* bacteria.

Primarily, a laboratory study was conducted to evaluate the efficacy of the Fenton and photo-Fenton treatments. After that, the same experimental conditions used in the laboratory bench setup were also applied to evaluate the efficacy of the pilot plant.

In general, the efficacy of a treatment applied in higher scale decreases in comparison with laboratory bench scale. This occurs because some modifications on the reactor project are usually necessary to reduce costs and to fit into the available space for installation. In this context, this study evaluated if the efficacy of the laboratory bench setup on sulfaquinoxaline oxidation, mineralization, and toxicity removal was equal, lower, or higher than the efficacy obtained using the pilot plant at the same experimental conditions. Even though, the setups used were different in relation to the reactor and lamp characteristics, it is worth knowing the versatility of the results obtained in this study.

## 2. Methods

### 2.1. Chemicals

Sulfaquinoxaline sodium salt (C_14_H_11_N_4_O_2_SNa, 95% purity), Table 1, was purchased from Sigma-Aldrich. Hydrogen peroxide (H_2_O_2_, 33% *w/v*), formic acid (CH_2_O_2_, 98%), and sulfuric acid (H_2_SO_4_, 98%) were from Panreac. Ferrous sulfate heptahydrate (FeSO_4_·7H_2_O) was purchased from Merck. Methanol (HPLC grade) was purchased from VWR. Ammonium metavanadate (NH_4_VO_3_, 99%) was from Honeywell Riedel-de-Haën. *Escherichia coli* and *Staphylococcus aureus* bacteria strains were ATCC 29213 and ATCC DH5α, respectively.

### 2.2. Experimental Setups

Oxidation assays were carried out using two different setups: laboratory bench (V = 2 L) and pilot plant (V = 15 L). The reactors and the lamps in each setup are described in detail below.

#### 2.2.1. Laboratory Bench

The photochemical reactor used to carry out the assays in the laboratory bench setup was a cylindrical batch made of borosilicate glass, double jacketed to maintain the temperature of the solution. A heating system was used to control the temperature of the water, that was continuously recirculated through the reactor during the assays. The total volume of the reactor was 2 L. The solution was continuously stirred using a magnetic stirrer, and the pH was monitored throughout the course of the reaction using a pH meter.

A high-pressure ultraviolet lamp of 300 W and spectral range of 450–550 nm (Ultra-Vitalux^®^, Osram, Barcelona, Spain) was used as the radiation source, simulating sunlight. The lamp was located at the top of the reactor, with a gap of 20 cm between the lamp and the solution surface. Actinometric measurements were performed for both reactors, laboratory bench, and pilot plant. It was used the potassium ferrioxalate method described by Murov et al. [30]. The incident photon flux was 1.18 × 10^−6^ Einstein s^−1^, corresponding to 0.31 J s^−1^. The energy density in the solutions ranged from 0 to 2232 J L^−1^.

#### 2.2.2. Pilot Plant

The automated pilot plant had a total volume of 15 L and it was composed of two main units of borosilicate glass. The first unit was a glass reservoir connected to a pump operating at a flow rate of 12 L min^−1^ to ensure complete mixing of the solution, as reported by our previous work [31]. The second unit was a tubular photochemical reactor (5.77 L) made of borosilicate glass with an internal cylinder where a fluorescent lamp of 36 W with spectral range of 350–450 nm (Philips Actinic BL TL-DK (Barcelona LED, Barcelona, Spain)) was inserted as the radiation source. This unit of the reactor was jacketed to maintain the temperature at 26 ± 2 °C. The incident photon flux at the photochemical reactor was 5.66 × 10^−7^ Einstein s^−1^, corresponding to 0.19 J s^−1^. The energy density at the solution ranged from 0 to 1824 J L^−1^.

A schematic representation of the experimental setup used at the laboratory bench and the pilot plant were reported in our previous works with more details, Pérez-Moya et al. [32] and Yamal-Turbay et al. [31], respectively.

### 2.3. Experimental Conditions and Analytical Methods

Experimental conditions for all assays conducted on both setups were the same: sulfaquinoxaline, iron (II), and hydrogen peroxide initial concentrations, temperature, pH of the solutions, and reaction times.

The initial concentration of the target compound is strictly related to the detection and quantification limits of the analytical equipment. To ensure the reliability of the results obtained in this study, the sulfaquinoxaline initial concentration was 25 mg L^−1^ prepared in distilled water. The pH of the solution was adjusted to 2.8 ± 0.1 using an H_2_SO_4_ solution (30%, *v*/*v*) before the addition of the Fenton reagents to the solution. In Fenton’s reaction, the pH adjustment is extremely important, because at pH value higher than 3, the Fe(III) precipitate as oxyhydroxides, which are insoluble and relatively did not react with the H_2_O_2_ [15]. At pH values below 2.5, the HO^•^ scavenging can occur because of the high H^+^ concentration; in addition to that, the less hydroxylated species are predominant at this pH value and they have low absorptivity. The pH slightly lower than 3 is the appropriate for Fenton’s reaction [15].

A study performed by Perini et al. [19] compared the level of oxidation and mineralization of paracetamol (PCT) by Fenton and photo-Fenton processes, using the same experimental conditions, varying the Fe(II) initial concentration between 5 and 10 mg L^−1^. Authors observed that, using 10 mg L^−1^ Fe(II), the time of reaction for complete oxidation of PCT reduced from 10 min to 2.5 min, endorsing that high catalyst concentration was a properly initial concentration for improvement of the process efficiency. Thus, the iron concentration was fixed at 10 mg L^−1^ because this is the maximum permissible value for wastewaters, according to Spanish legislation [33], and is lower than the limit established by Brazilian legislation (15 mg L^−1^) [34].

The stoichiometric hydrogen peroxide concentration required to achieve total mineralization of 25 mg L^−1^ SQX to CO_2_, H_2_O, and inorganic ions, is shown in Equation (7). According to this stoichiometric relation, 118.7 mg L^−1^ H_2_O_2_ is the minimum concentration required to achieve total mineralization of SQX.

(7) $$C_{14}H_{11}N_{4}O_{2}SNa\;+\;45H_{2}O_{2}\;\to 14CO_{2\;}+\;48H_{2}O\;+4HNO_{3}+NaHSO_{4}$$

Throughout the reaction time on both experimental systems, laboratory bench and pilot plant, samples were collected for monitoring SQX concentration, the mineralization level of the SQX and the reaction products, the residual concentration of H_2_O_2_, and the residual toxicity. The reaction times were as follows: 0, 2, 5, 10, 15, 20, 25, 30, 45, 60, 90, 120, 150, 180, 210, and 240 min.

The sulfaquinoxaline concentration was determined by high performance liquid chromatography (HPLC) analytical technique, using an Agilent 1200 instrument equipped with a photodiode array detector, set at 250 nm. Methanol and 0.1% (*v*/*v*) formic acid (55:45, *v*/*v*) were used as the mobile phase, and the stationary phase was an Akady HPLC Ultrabase 5 C-18 column (150 × 4.6 mm, 5 µm), kept at 25 °C. The injection volume was 20 µL and the flow rate was 1.0 mL min^−1^. Methanol was used to dilute the samples (by 50% of the original concentration) before injection into the HPLC. This solvent acts as a hydroxyl radical scavenger [35] and was used to interrupt the oxidation reaction. The limits of detection and quantification were 0.05 mg L^−1^ and 0.25 mg L^−1^ SQX, respectively.

The mineralization of the sulfaquinoxaline and of the reaction products were determined using a total organic carbon analyzer (TOC-V_CSH/CSN_, Shimadzu, Kyoto, Japan) with a quantification limit of 0.5 mg L^−1^ for organic carbon and 1.0 mg L^−1^ for total carbon. Because of the total carbon quantification limit, the initial concentration of SQX could not be lower than 25 mg L^−1^; at this concentration, the theoretical total carbon was 13.04 mg L^−1^, which means that the maximum TOC removal that could be observed in this study would be 92%. The samples submitted to TOC’s evaluation were kept at a low temperature and analyzed immediately after sampling.

The residual H_2_O_2_ concentrations were determined by the ammonium metavanadate colorimetric method, as described by Nogueira et al. [36].

Residual toxicity was evaluated using two bacteria as organism test, the Gram-positive *Staphylococcus aureus* (ATCC 29213), and the Gram-negative *Escherichia coli* (ATCC DH5α). Ultrapure water was used as a control and SQX solutions (0.1, 1, 10, 100, 1000, and 10,000 mg L^−1^), were used to evaluate the bacterial growth inhibition caused by sulfaquinoxaline.

The assays were performed using a microtiter plate of 96 wells. Each well was filled with 50 µL of bacteria suspension in Luria–Bertani medium (corresponding to 10^3^ CFU mL^−1^) and 200 µL of the sample (initial sample and the samples subjected to the oxidative assays). The plates were incubated at 37 °C for 24 h, followed by measurement of the absorbance at 595 nm using a microplate reader (EZ Read 400, Biochrom Ltd., Cambridge, UK).

The toxicity assays were performed in duplicate and the results were expressed by the percentage of growth inhibition (assessed by comparing the differences between the absorption values of the control and the samples subjected to the oxidation processes).

The toxicity of the H_2_O_2_ to the organism tests was evaluated and it was observed that, at concentrations lower than 2.0 mg L^−1^ H_2_O_2_, the bacterial growth was not damaged. The toxicity assay was performed collecting samples corresponding to the time after the H_2_O_2_ was exhausted (less than 2 mg L^−1^) and the TOC measurement reached a plateau (data shown correspond to 120 min reaction time).

### 2.4. Design of Experiments (DOE)

A design of experiments was used in this study to evaluate the significance of each variable (hydrogen peroxide concentrations, presence or absence of radiation source (ON and OFF), and its interaction for both setups: laboratory bench and pilot plant).

Stoichiometric H_2_O_2_ concentration (118.7 mg L^−1^) was selected as the lower level (−1), and double amount 237.3 mg L^−1^ as the higher level (1). Table 2 describes all experimental conditions and shows the H_2_O_2_ concentration range (94.1 to 261.9 mg L^−1^). The design included four central points (E–H) and two star points (I–J). All the conditions summarized in Table 2 were studied at the laboratory bench setup, followed by testing the best conditions using the pilot plant. All the assays were carried out at least twice.

The response factors were oxidation and mineralization of sulfaquinoxaline and the residual toxicity of the solutions subjected to oxidation processes.

From this experimental design, it is expected to get the following information: if it is possible to oxidize or/and mineralize SQX using Fenton and photo-Fenton processes, if it is possible to decrease the toxicity of the solutions, and which of the studied variables were significant in the proposed setups.

### 2.5. Proposed Model

A semi-empirical model already tested in our previous works [19,37] was used to characterize the performance of the processes studied at the laboratory bench and pilot plant.

The evolution of the *SQX* concentration can be described by Equation (8).

(8) $$\left[SQX\right]={\left[SQX\right]}^{\infty }+\left(SQX^{0}-SQX^{\infty }\right)\times e^{-kt}$$

where *SQX*^0^ is the initial sulfaquinoxaline concentration, *SQX*^∞^ is the sulfaquinoxaline concentration when maximum degradation was obtained, *k* is the degradation rate, and t is the reaction time. The conversion (*ξ*) can be described by Equation (9).

(9) $$\xi =\xi^{max}\left(1-e^{-kt}\right)$$

where *ξ^max^* is the maximum conversion and can be defined by Equation (10).

(10) $$\xi^{max}=\frac{{\left[SQX\right]}^{0}-{\left[SQX\right]}^{\infty }}{{\left[SQX\right]}^{0}}$$

Hence, the oxidation performance can be characterized by determining the two parameters of the model, *ξ^max^* (or (*SQX*)^∞^) and *k*, which can be obtained by fitting the model to the experimental data, using the least squares criterion.

## 3. Results and Discussion

Oxidation of sulfaquinoxaline by Fenton and photo-Fenton processes was evaluated at the following conditions: SQX initial concentration of 25 mg L^−1^, Fe(II) of 10 mg L^−1^, temperature of the solution at 26 ± 2 °C, and pH 2.8 ± 0.1. The variables under study were established for each assay following Table 2.

From this point on, experiments were named according to the following nomenclature of the variables: X_(Y_Z), where X is the assay, Y is the H_2_O_2_ concentration in mg L^−1^, and Z is the presence (ON) or absence of radiation (OFF).

### 3.1. Photolysis, Peroxidation, and Photoperoxidation of Sulfaquinoxaline

Blank assays were performed to evaluate the effects of the photolysis, peroxidation and photoperoxidation processes on SQX oxidation. The process of photolysis was not effective to oxidize and mineralize the sulfaquinoxaline on both setups, laboratory bench (UV lamp 350–400 nm) and pilot plant (UV lamp 400–550 nm). Less than 5% of SQX oxidation and mineralization was obtained by these setups.

The processes of peroxidation and photoperoxidation were carried out using 237.3 mg L^−1^ H_2_O_2_ (two times the stoichiometric dose), in the absence and presence of UV radiation. Results were equal in presence or absence of radiation for both setups. Due to the proper radiation emission to ensure the UV/H_2_O_2_ process is 254 nm, as the previous studies that Liao et al. [22] reported, SQX (10 mg L^−1^) using 200 mg L^−1^ H_2_O_2,_ and a UV lamp of 20 W/254 nm, achieved an SQX oxidation higher than 90%. However, in the present study, the lamps used were not the appropriate to the UV/H_2_O_2_ process, and they fit better with the photo-Fenton appropriate wavelength. Peroxidation or photoperoxidation assays resulted in 18% SQX oxidation and no mineralization at the laboratory bench and at the pilot plant slightly better results were obtained.

### 3.2. Sulfaquinoxaline Oxidation

The Fenton and photo-Fenton processes provided efficient oxidation of SQX present in the solutions at the following conditions: SQX initial concentration of 25 mg L^−1^, 10 mg L^−1^ Fe(II), temperature at 26 ± 2 °C, and pH 2.8 ± 0.1.

The efficiency of the oxidation process was evaluated as a function of different concentrations of H_2_O_2_ and the presence or absence of UV radiation. The H_2_O_2_ concentrations used in the assays corresponded to the stoichiometric dose (118.7 mg L^−1^), as described in Section 2.2, a central concentration (178.0 mg L^−1^), and double of the stoichiometric dose (237.3 mg L^−1^).

HPLC analysis was used to monitor the decay of the SQX concentration from its initial value (*SQX*^0^) at t = 0. The experimental data were used to fit the previous proposed model, using the least squares criterion.

The regression coefficients (R^2^) were calculated to indicate the quality of the model fits. The R^2^ values were between 0.90 and 0.99 for both scales studied, reflecting significant relations between the experimental data and the models.

**Figure 1:**
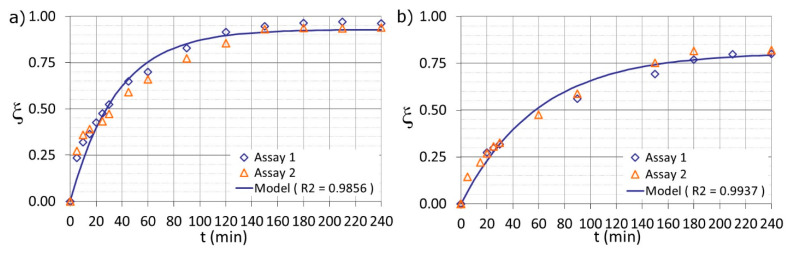
Experimental data from the central points of the DOE fit the pseudo-first order model for (**a**) photo-Fenton (Assays E-F) and (**b**) Fenton (Assays G-H) and processes at the laboratory bench setup.

Degradation rates (*k*) obtained at the laboratory bench ranged from 0.017 to 0.031 min^−1^, while rates from 0.065 to 0.108 min^−1^ were obtained for the pilot plant. The model fitting of the experimental design central points: E, F (178.0_ON) and G, H (178.0_OFF) for the laboratory bench study is shown in Figure 1.

The model parameters for the Fenton process (Figure 1a) were *k* = 0.017 min^−1^ and *ξ^max^* = 0.81, and for the photo-Fenton process (Figure 1b) were *k* = 0.027 min^−1^ and *ξ^max^* = 0.93. Each experiment was carried out in duplicate (Assay 1 and Assay 2). The final model was obtained of the fitting of the data in duplicate.

As shown in Figure 1, SQX was removed efficiently by Fenton and photo-Fenton processes. After 90 min of reaction, the maximum oxidation was 57% and 80% (laboratory bench) and 79% and 93% (pilot plant) for Fenton and photo-Fenton, respectively. The use of radiation clearly increased the efficiency of the process in terms of the maximum SQX degradation and the decay rate. Moreover, the study permitted to highlight that Fenton process was capable to oxidize the SQX solution.

**Figure 2:**
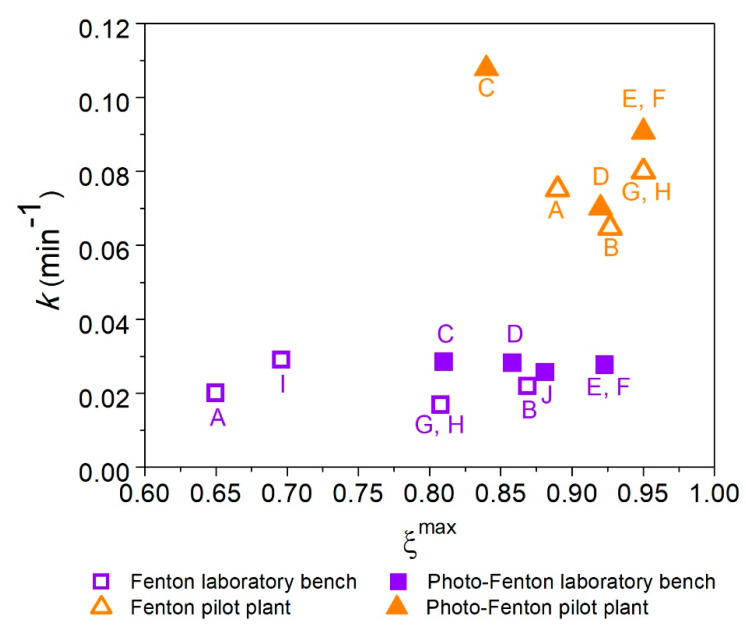
Degradation rates as a function of maximum conversion (*ξ^max^*) obtained on both setups: laboratory bench and pilot plant.

The efficiencies of the processes at the different setups (laboratory bench and pilot plant) were evaluated using as performance parameters the degradation rate (*k*, min^−1^), and the maximum conversion (*ξ^max^*). The results obtained using a pseudo-first order model to determine these parameters are shown in Figure 2. The best condition should have a high degradation rate and *ξ^max^* close to one.

The oxidation reactions were faster for the pilot plant than for the laboratory bench for both processes, as shown in Figure 2. The initial concentration of sulfaquinoxaline (25 mg L^−1^) decreased by 50% after approximately 60 min of reaction in the laboratory bench, while the same oxidation was obtained after 10 min in the pilot plant for both processes and conditions evaluated. These results can be explained by the additional oxidation provided by oxygenation of the solution in the pilot plant. This oxygen was generated by the recirculation of the solution at a high flow rate (12 L min^−1^), and improved the system efficiency by increasing the degradation rate and producing more oxidant species during the process [15].

The maximum conversion and the highest degradation rate of SQX were obtained in the photo-Fenton assays, as shown in Figure 2. The assays conditions related to the central points of the experimental design, E and F (178.0_ON), were more effective on SQX oxidation, that is, the use of an intermediate oxidant concentration resulted in higher degradation rates and high *ξ^max^* values for both setups studied.

Except the laboratory bench assays A, and B, which presented very similar degradation rates (0.0306 and 0.0285 min^−1^, respectively), all the photo-Fenton assays showed higher degradation rates, compared to the Fenton process. This indicated that the reagent concentrations used in the photo-Fenton process were satisfactory and that HO^•^ scavenging or competition for the photons provided by the radiation source did not occur, so the efficiency of the process was not diminished. The laboratory bench photo-Fenton assays C, D, E, and F presented very similar degradation rates (0.0285, 0.0282, 0.0277, and 0.0277 min^−1^, respectively), indicating that a higher H_2_O_2_ concentration did not directly lead to a gain in efficiency.

The degradation rates obtained in the pilot plant were two-fold higher than those achieved at the laboratory bench, considering assays A (118.7_OFF), B (237.3_OFF), and D (237.3_ON) of the experimental design. The values of *k* obtained for assays C (118.7_ON), E (178.0_ON), and F (178.0_ON) in the pilot plant were three times higher than achieved at the laboratory scale, while the values for pilot plant assays G and H (178.0_OFF) were four times higher than for the laboratory bench. This probably happened because, during the recirculation process, the solution oxygenation occurred.

It is interesting to highlight the results of pilot plant assay C (118.7_ON), the one with higher degradation rate (Figure 2). Clearly, 118.7 mg L^−1^ was an adequate hydrogen peroxide amount, so the mass ratio of Fenton reagent H_2_O_2_:Fe(II) (11.9) and the mass ratio H_2_O_2_:SQX (4.76) were appropriate. As a result, assay C (118.7_ON) was the one with the faster initial conversion. However, after exhaustion of the H_2_O_2_, the maximum conversion (*ξ_max_* = 0.81) was lower than assays E and F (178.0_ON) (*ξ_max_* = 0.92). This suggests that a better experimental condition might be applied to start the assay. The stoichiometric H_2_O_2_ concentration of 118.7 mg L^−1^ could be used in order to achieve faster initial conversion, then adding extra H_2_O_2_ dosage during the course of the assay, in order to keep the initial efficiency of the assay C (118.7_ON). The result of this H_2_O_2_ addition (H_2_O_2_ dosage) would increase the efficiency of the process, because of better use of the Fenton reagent, as already reported by other authors [38].

### 3.3. Mineralization

The mineralization of the organic compounds, SQX, and the reaction products was evaluated by monitoring the concentration of total organic carbon. In the case of the Fenton process, the mineralization obtained at the laboratory bench ranged from 0 to 24%, while for the pilot plant the values ranged from 30 to 34%. For the photo-Fenton process, the performance at the laboratory bench was from 41 to 56% and at the pilot plant from 40 to 56%. Based on the results obtained in this study, both Fenton and photo-Fenton processes were more effective to mineralize sulfaquinoxaline than the UV/H_2_O_2_ process reported by Liao et al. [22]. They reported 10% sulfaquinoxaline (10 mg L^−1^, 0.1 L) mineralization after using 80 mg L^−1^ H_2_O_2_ and UV dose of 1476 mJ cm^−2^ and 30% mineralization after 4428 mJ cm^−2^. Fenton and photo-Fenton processes with 178.0 mg L^−1^ hydrogen peroxide mineralized 30 and 50% (respectively) of sulfaquinoxaline (25 mg L^−1^, 15 L). Clearly, the photo-Fenton process reached higher oxidation and mineralization of sulfaquinoxaline using lower concentration ratio of H_2_O_2_:SQX = 7 when compared to the UV/H_2_O_2_ process with an H_2_O_2_:SQX concentration ratio of 8. Comparing UV/H_2_O_2_ and Fenton processes, similar results in relation to mineralization were attained, even though it is important to highlight that Fenton process is easier and cheaper to implement than UV/H_2_O_2_ process, because of its independence of a radiation source which helps to decrease the energy demand and the requirements for the reactor setup.

**Figure 3:**
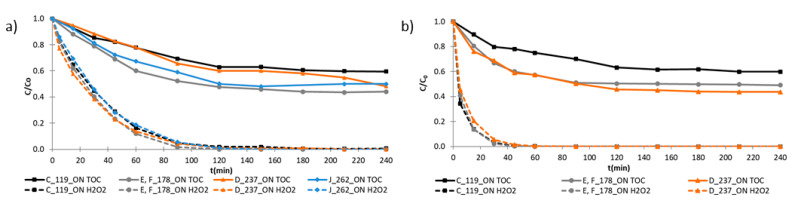
Mineralization (continuous line) and hydrogen peroxide consumption (discontinuous line) along the reaction time of photo-Fenton process at the (**a**) laboratory bench and (**b**) pilot plant.

Figure 3 shows the evolution of the residual concentration of total organic carbon (continuous line) and hydrogen peroxide (discontinuous line) for photo-Fenton process using the laboratory bench (Figure 3a) and the pilot plant (Figure 3b) setups.

The different mineralization rates obtained in the presence of UV radiation were related to the amount of hydrogen peroxide provided in the process. Photo-Fenton process, with 178.0 mg L^−1^ H_2_O_2_ (assays E and F) (Figure 3a,b), mineralized around 50% either for pilot plant or 56% for laboratory bench setup, corresponding to a sulfaquinoxaline oxidation over 90% for both studied setups. There was no significant benefit of increasing the H_2_O_2_ concentration above 178.0 mg L^−1^, even in the pilot plant, especially considering economic factors. However, the increase of this oxidant concentration to slightly above the stoichiometric value (118.7 mg L^−1^) was useful.

The significance of the UV radiation was evaluated by comparing the mineralization efficiencies of Fenton and photo-Fenton processes. The use of UV radiation increased mineralization by 28–56% in the laboratory bench and 6–26% in the pilot plant. It is possible that additional oxidant was present in the pilot plant since its configuration and the high mixing rate helped to introduce oxygen into the system, which contributed to the mineralization with (photo-Fenton) and without (Fenton) UV radiation. The laboratory setup did not receive any significant concentration of additional oxidants, so consequently, the main radicals present in the solution were provided from the Fenton and photo-Fenton reactions, making the difference between the processes more evident.

The photo-Fenton results obtained in this study, for both setups (Figure 3), showed that the residual H_2_O_2_ concentration could be highlighted as a limiting factor for mineralization, because the mineralization reached a plateau after complete H_2_O_2_ consumption at 120 min reaction time. However, in the Fenton assays conducted using the laboratory system (A, B, G, H, and I), there was the presence of residual H_2_O_2_ after this time, with no observed increase in mineralization and degradation. This probably happened because in the Fenton process, there is the absence of UV radiation, which is fundamental to reduce Fe(III) to Fe(II) and the residual H_2_O_2_ did not react with the Fe(II) to produce hydroxyl radicals.

### 3.4. Toxicity of the Solutions

It is generally acknowledged that, in some cases, advanced oxidation processes are unable to achieve complete mineralization of the target compound, and consequently oxidation products can be formed. These oxidation products are not desirable, because they can present toxicity greater than the parent compound. Consequently, it is necessary to evaluate the toxicity of the samples along the reaction time.

**Figure 4:**
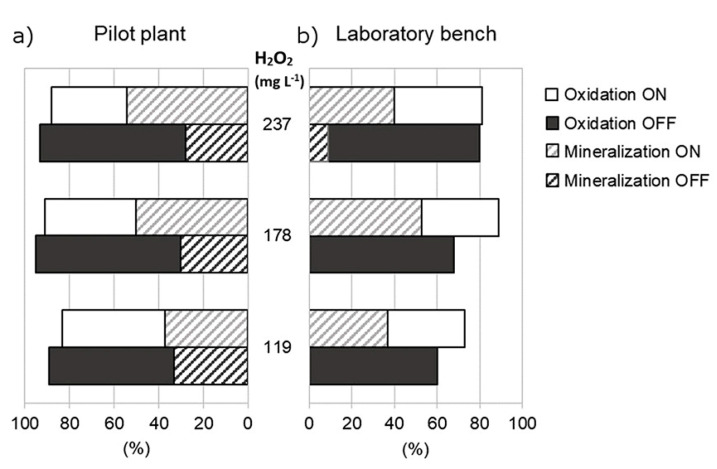
Oxidation of sulfaquinoxaline and mineralization after the reaction time of 120 min by the Fenton (OFF) and photo-Fenton (ON) processes at the (**a**) pilot plant and (**b**) laboratory bench.

To understand the contributions of the parent compound, and its degradation products to the residual toxicity of the solutions subjected to degradation processes, Figure 4 shows the SQX (a) oxidation and (b) mineralization for both setups studied after reaching the plateau related to total organic carbon evolution (120 min reaction time).

High sulfaquinoxaline oxidation combined with low mineralization is probably indicative of the oxidation products generation during the reaction. For this reason, the residual toxicity of the solutions subjected to the oxidation processes was evaluated along with sulfaquinoxaline oxidation and mineralization. Photo-Fenton process at laboratory bench after 120 min of reaction oxidized between 73% and 89% SQX at the assays C (118.7_ON), E (178.0_ON), and F (178.0_ON) (Figure 4). For these same conditions, mineralization reached around 37–53% (Figure 4). These results suggested that degradation products were probably formed.

Toxicity assays using *E. coli* and *S. aureus* were carried out to evaluate the toxicity of the solutions after oxidation. Preliminary assays were undertaken to assess the toxicity due to H_2_O_2_ at concentrations ranging from 0.46 to 237 mg L^−1^. Approximately 90% bacterial growth inhibition was observed for both microorganisms using H_2_O_2_ concentrations between 3.7 and 237.3 mg L^−1^, while normal growth was observed using H_2_O_2_ concentrations below 2 mg L^−1^.

Toxicity was performed for solutions taken to the reaction time of 120 min, in which the H_2_O_2_ was completely consumed or its concentration was under 2 mg L^−1^. As previously explained, H_2_O_2_ concentration smaller than 2 mg L^−1^ did not interfere on the normal bacterial growth. Thus, it was not necessary to quench the residual H_2_O_2_ of the samples before conducting the toxicity assay.

The lowest concentrations of sulfaquinoxaline that can cause any effect to the *Daphnia magna*, *Pseudokirchneriella subcapitata*, *Scenedesmus dimorphus*, *Synecococcus leopoliensis*, and *Lemna gibba* test microrganisms were 1.563, 0.078, 0.039, 0.156, and 5 mg L^−1^, respectively [8]. Dalla Bona et al. [39] obtained SQX EC_50_ (concentration of the drug that induces half of the maximum effect) values of 129.3 and 84.46 mg L^−1^ for *Daphnia magna* and *Daphnia curvirostris*, respectively. In this study, the growth inhibition of *E. coli* and *S. aureus* when in contact with 100 mg L^−1^ SQX were 40% and 45%, respectively, which is in accordance with the values reported in literature.

**Figure 5:**
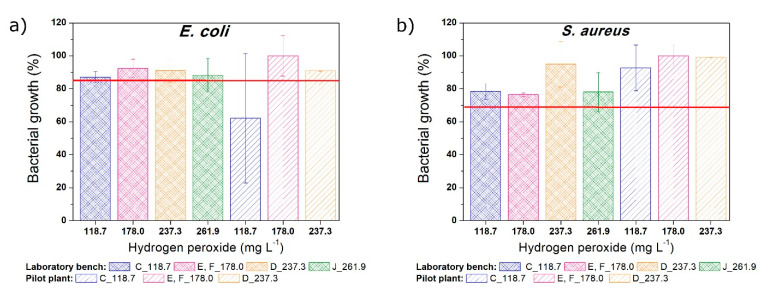
Growth inhibition of (**a**) *Escherichia coli* and (**b**) *Staphylococcus aureus* bacteria caused by the toxicity of the solutions subjected to photo-Fenton process at the laboratory bench and pilot plant.

To evaluate the toxicity of the SQX solutions, the bacterial growth inhibition caused by 25 mg L^−1^ SQX (initial concentration of this study) was determined. The growth inhibition of *Escherichia coli* (15%) and *Staphylococcus aureus* (32%) was represented in the Figure 5 using a red line.

The toxicity at 120 min of reaction is shown in Figure 5 for the different experimental conditions tested (using different H_2_O_2_ concentrations under radiation) and the two microorganisms (*E. coli* and *S. aureus*). The red lines indicated the relative growth of the bacteria in the presence of 25 mg L^−1^ SQX. Hence, where the relative growth surpasses the red line, the solution was less toxic than the parent compound solution, in several cases reaching values close to 100%, compared to the controls. However, when the bacterial growth was lower than the red line, the oxidation process increased the solution toxicity.

The solutions subjected to photo-Fenton oxidation process at laboratory bench setup did not interfere in the bacterial growth. The growth of *E. coli* and *S. aureus* bacteria were higher than the initial inhibition (above the red line) (Figure 5a,b). *S. aureus* bacterium growth did not decrease after the oxidation processes evaluated for both setups, pilot plant and laboratory bench. However, using the *E. coli* bacterium (Figure 5a) as test organism at the pilot plant, the assay C (118.7_ON) was the exception result, and the bacterial growth decreased if compared to the initial solution. Probably, it happened because of the low hydrogen peroxide concentration used in this specific assay; in this case, some degradation products were formed, and they were not mineralized. Further tests should be performed to better assess this specific result. Moreover, the high standard deviation of the assay C using the *E. coli* bacterium (performed in duplicate) requires a careful conclusion.

In contrast, it can be seen from Figure 5b (test with *S. aureus* bacterium) that the possible degradation products formed at 120 min of reaction at the pilot plant (even the C assay) were clearly less toxic than the initial SQX solution.

Fenton assays after 120 min of reaction presented residual hydrogen peroxide (around 20–50 mg L^−1^). Even though, toxicity tests were carried out (data not shown) without quenching the residual hydrogen peroxide. The toxicity results for both setups studied (laboratory bench and pilot plant) showed that the solution subjected to this process presented lower toxicity than the parent compound. To clearly identify the toxicity value in Fenton samples, deeper study is required. This preliminary study helps only to know that, after Fenton treatment, toxicity did not increase.

Finally, it is worth mentioning other studies [40] that confirmed the acute toxicities decrease after sulfaquinoxaline treated by UV/H_2_O_2_/Fe(II). Thus, it might indicate that the treatment was a safe AOP to remove the sulfonamides in aqueous.

### 3.5. Experimental Design Results

The influence of different variables (presence or absence of the radiation source and hydrogen peroxide concentration) on the Fenton and photo-Fenton processes were evaluated for four different reaction times (30, 60, 120, and 240 min) and two different response factors: sulfaquinoxaline oxidation and mineralization. The statistical analyses were performed using the laboratory bench and pilot plant results.

Pareto charts were used to determine the effect of each variable on the response factors, and the results obtained for the laboratory bench and the pilot plant were summarized in Table 3. The numbers indicate notable effects, and the order of significance according to the factor or its interaction.

The absence of any number in Table 3, such as in the case of SQX oxidation at 30 min of reaction in the laboratory bench, indicated that this response factor was not significantly affected by the presence or absence of radiation, by the hydrogen peroxide concentration, or by the interaction of the two variables at the timeframe studied and variables spam. Hence, the significance of the studied factors and their interaction could be neglected, for this specific reaction time. The same conclusions can be reached in the case of the pilot plant for 30 min of reaction time.

In the laboratory bench setup, the presence or absence of radiation was the only significant variable affecting sulfaquinoxaline mineralization, for all reaction times evaluated (Table 3). After 60 min of reaction, the effect of radiation or not was significant, and for the longest reaction time (240 min), the interaction of the factors (ON/OFF and H_2_O_2_ concentration) was significant for the oxidation of SQX.

For the pilot plant, the studied variables were more significant to the mineralization and oxidation of sulfaquinoxaline when compared to the laboratory bench setup. The interaction of the variables and the H_2_O_2_ concentration became more significant at longer reaction times (240 min). In the case of mineralization, all the factors were significant at the longest reaction time, following the decreasing order: presence or absence of radiation, interaction of the variables, and H_2_O_2_ concentration. For shorter reaction times (30, 60, and 120 min), the two variables, as well as their interaction, were not significant for SQX oxidation, under the conditions studied. However, at 240 min, the presence or absence of radiation was significant, as was the H_2_O_2_ concentration.

Minitab^®^ software was used to produce a simplified (without physical significance) model representing the behaviors of the systems in the timeframe studied, which could be used to predict the performance obtained using values that were not tested. Models were obtained for each response factor at different reaction times. Table 4 summarizes the models for sulfaquinoxaline oxidation and mineralization after 240 min reaction time for the laboratory bench and the pilot plant. The response factor (SQX oxidation or mineralization) is represented by *y* in the model equation, and *x* represents the hydrogen peroxide concentration.

**Figure 6:**
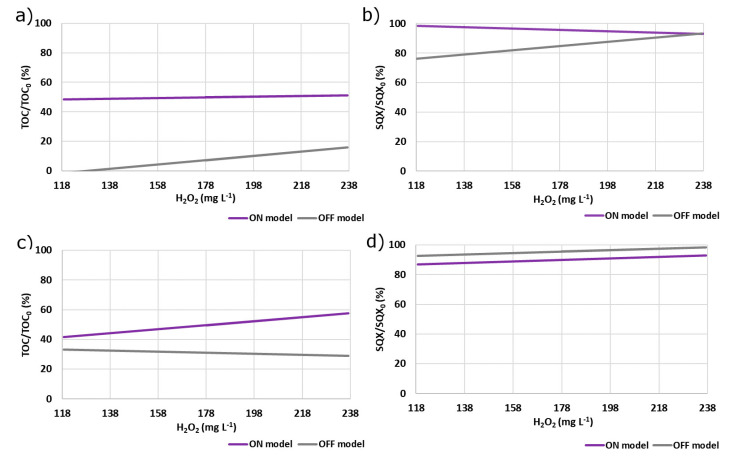
Model fitting of the experimental data after 240 min of reaction using Fenton (OFF) and photo-Fenton (ON) processes. The response factors (mineralization and sulfaquinoxaline oxidation) were evaluated to the laboratory bench (**a**,**b**) and to the pilot plant (**c**,**d**), respectively.

In the case of the laboratory bench, the mineralization model for the photo-Fenton process is shown in Figure 6a. The H_2_O_2_ concentration had no significant effect on mineralization, within the timeframe studied, while the opposite was observed for the Fenton process, where mineralization increased as the H_2_O_2_ concentration increased. The same trend was observed for SQX oxidation (Figure 6b), with oxidation by the Fenton process increasing as the H_2_O_2_ concentration was increased. The efficiency of the photo-Fenton process did not increase as the oxidant concentration increased, probably due to the competition between SQX and hydrogen peroxide for the photons provided by the lamp, which decreased the oxidation efficiency.

The mineralization results obtained for the Fenton and photo-Fenton processes using the pilot plant are shown in Figure 6c, from which the model provided a satisfactory fit to the experimental data. In the case of the photo-Fenton process, mineralization increased as the H_2_O_2_ concentration increased, while the opposite occurred for the Fenton process, with mineralization decreasing as the H_2_O_2_ concentration increased. Fenton and photo-Fenton present the same trend for SQX oxidation (Figure 6d), that increases as the H_2_O_2_ concentration also increased. Good agreement between the experimental data and the model was also observed as the R^2^ index in Table 4 illustrates. The results of the statistical analysis agreed with the results previously discussed on Section 3.2 and Section 3.3.

Further work should focus on the treatment of more complex antibiotic solutions (water matrix effect, different TOC concentrations and pollutants, etc.) together with the possibility of using dosage strategies in order to reach a more efficient process design.

## 4. Conclusions

Photo-Fenton and Fenton processes provided effective oxidation of sulfaquinoxaline, and the presence of a radiation source enhanced mineralization of the organic compounds. However, the study permitted to highlight that Fenton process was capable to oxidize the SQX and attain a degree of mineralization (30%) similar to the other process published in the specialized literature (UV/H_2_O_2_).

The results obtained at the laboratory bench and pilot plant setups were similar in terms of SQX oxidation. The photo-Fenton process using 178.0 mg L^−1^ H_2_O_2_ was highlighted as the best condition studied, and for both setups, mineralization was higher than 50% and more than 90% of the initial SQX concentration was oxidized after 120 min reaction time. Use of a higher H_2_O_2_ concentration (237.3 mg L^−1^) did not significantly improve the efficiency in terms of increasing oxidation and mineralization, or decrease the toxicity of the solutions, while a lower H_2_O_2_ concentration (118.7 mg L^−1^) clearly reduced the oxidation and mineralization efficiency.

The toxicity results revealed a slight difference between the microorganisms used, with *S. aureus* being more sensitive to the initial sulfaquinoxaline concentration than *E. coli*. The optimum photo-Fenton condition (178.0 mg L^−1^ H_2_O_2_) resulted in no growth inhibition in the pilot plant and laboratory bench for both bacteria. The degradation products formed after partial mineralization of the solutions did not affect the growth of the *S. aureus* bacterium and did not presented synergistic effect between then.

Models of sulfaquinoxaline oxidation and mineralization were proposed to represent the behavior of the setups (laboratory bench and pilot plant) and predict their performance using other conditions in the studied range but that were not tested by this study. Correlation coefficients higher than 93% indicated good fits of the models.

The significant variables for sulfaquinoxaline mineralization at the pilot plant were (in descending order) the presence or absence of radiation, the interaction between the variables, and the H_2_O_2_ concentration. In the laboratory bench setup, only the presence or absence of radiation was significant. In both setups, the presence or absence of radiation was equally important for sulfaquinoxaline oxidation. However, the interaction between the variables was more important at the laboratory bench and the H_2_O_2_ concentration was more significant using the pilot plant.

In general, the pilot plant was considered more efficient than the laboratory bench because its setup configuration provided an additional oxygenation, ensured full utilization of the photons, and complete homogeneity of the solutions, resulting in a faster degradation rate and higher maximum sulfaquinoxaline conversion.

The differences between the mineralization obtained applying Fenton and photo-Fenton processes were more significant than the oxidation. At the pilot plant, applying the Fenton process, 34% mineralization was obtained using 118.7 mg L^−1^ H_2_O_2_ (assay A) and the use of UV radiation (assay C) increased the mineralization to 40%.

## Figures and Tables

**Table 1:** Sulfaquinoxaline chemical structure and molar mass.

Name (Abbreviation)	Chemical Structure	Molecular Mass (g mol^−^^1^)
Sulfaquinoxaline sodium salt (SQX)	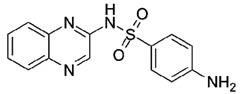	332.32

**Table 2:** Experimental conditions for sulfaquinoxaline oxidation by Fenton and photo-Fenton processes.

Assay	Coded Values		Variables	
	H_2_O_2_	UV Radiation	H_2_O_2_ (mg L^−1^)	UV Radiation
A	−1	−1	118.7	OFF
B	1	−1	237.3	OFF
C	−1	1	118.7	ON
D	1	1	237.3	ON
E	0	1	178.0	ON
F	0	1	178.0	ON
G	0	−1	178.0	OFF
H	0	−1	178.0	OFF
I	−1.414	−1	94.1	OFF
J	1.414	1	261.9	ON

**Table 3:** Effects of the variables (presence or absence of radiation (ON/OFF), H_2_O_2_ concentration, and their interaction) on the response factors (mineralization and sulfaquinoxaline oxidation).

Response Factor	Reaction Time (min)	Laboratory Bench			Pilot Plant		
		ON/OFF	H_2_O_2_	Interaction	ON/OFF	H_2_O_2_	Interaction
Mineralization	30	1					
	60	1			1		
	120	1			1		2
	240	1			1	3	2
Oxidation	30						
	60	1					
	120	1					
	240	1		2	1	2	

**Table 4:** Summary of models for sulfaquinoxaline oxidation and mineralization after 240 min reaction time using the Fenton and photo-Fenton processes in the laboratory bench and the pilot plant.

Setups	UV-Visible	SQX Oxidation	R^2^	Mineralization	R^2^
Laboratory bench	OFF	*y* = 0.1434*x* + 59.27	98.00%	*y* = 0.1441*x* − 18.44	93.72%
	ON	*y* = −0.0472*x* + 104.18	98.00%	*y* = 0.0237*x* + 45.58	93.72%
Pilot plant	OFF	*y* = 0.05056*x* + 86.50	95.07%	*y* = −0.0337*x* + 37.00	98.44%
	ON	*y* = 0.05056*x* + 81.00	95.07%	*y* = 0.1348*x* + 25.50	98.44%
